# Use of a 4 mm Amplatzer Vascular Plug II in the treatment of a renal arteriovenous fistula: a case report

**DOI:** 10.1186/s42155-021-00229-3

**Published:** 2021-05-14

**Authors:** Davide Castellano, Andrea Boghi, Chiara Comelli, Luca Di Maggio, Daniele Savio

**Affiliations:** Department of Interventional Radiology and Neuroradiology, Ospedale San Giovanni Bosco, Torino, Italy

**Keywords:** Congenital renal arteriovenous fistula, Percutaneous embolization, Amplatzer vascular plug II

## Abstract

**Background:**

We report the use of a 4 mm vascular Amplatzer for the occlusion of a renal arterovenous fistula between the renal artery, at the hylum trifurcation point, and an aneurismatic vein draining into the main renal vein, where there was no possibility to use any other device from the venous side, because of the diameter and the high flow, neither from the arterious side without sacrificing lobar branches. The device was implanted at the exact point of communication, like a patent foramen ovale occluder, with the distal disc into the artery lumen and the other two proximal discs into the venous side.

**Case presentation:**

A 34-years-old Caucasian woman suffered several episodes of paroxysmal supraventricular tachycardia associated with dyspnoea, after the onset of post-pregnancy hypertension. She underwent CTA, spectral Doppler sonography and angiography which showed a renal arteriovenous fistula (RAVF) between the renal artery, at the hylum trifurcation point, and an extremely ectatic vein draining into the main renal vein of the right kidney.

With both arterial and venous access, the RAVF was selectively embolized using a 4 × 6 mm Amplatzer Vascular Plug II, released into the communication between artery and vein ensuring the patency of vessels involved.

The RAVF was almost completely excluded and the hemodynamic effects associated were also corrected.

**Conclusions:**

The use of this device, though in an alternative way, allowed the exclusion of the high flow A-V fistula without sacrificing any parent renal vessel and preserving the renal function.

## Background

Renal arteriovenous fistulas (RAVFs) are rare (Campbell JE, Davis C, DeFade BP, Tierney JP, Stone PA., 2009). They may represent an acquired non traumatic shunt (due to a neoplasm, inflammation, renal artery aneurysm, fibromuscular dysplasia or arterial dissection) (Marumo et al., 2016), a post-traumatic shunt (i.e. post biopsy) (Carrafiello et al., 2011), a congenital shunt (20%) or an idiopathic shunt (3%) (Campbell JE, Davis C, DeFade BP, Tierney JP, Stone PA., 2009; Carrafiello et al., 2011). Renal AVFs correspond to a type I shunt according to the angiographic classification of AV malformations by Cho et al. (Cho et al., 2006) and their treatment could be more challenging compared to the other types due to the bigger size of vessels involved, the direct communication between artery and vein and the consequent high flow (Marumo et al., 2016).

AVFs present with a variety of symptoms, including high output cardiac state (HOS, featuring tachycardia, heart congestion and dyspnoea), refractory hypertension, hematuria, abdominal pain and flank bruits (Marumo et al., 2016).

The usual treatment has been surgical, consisting in legation of arterial feeder, nephrectomy or partial nephrectomy (Carrafiello et al., 2011; Cho & Stanley, 1978), but endovascular treatment can now be considered as an alternative (Chatziioannou et al., 2005).

We present the case of a woman with HOS caused by a renal arteriovenous fistula (RAVF) treated with percutaneous embolization using a 4 mm Amplatzer Vascular Plug II.

In this patient the arteriovenous communication was between the renal artery, at the hylum trifurcation point, and an aneurismatic vein draining into the main renal vein, where there was no possibility to use any other device from the venous side, because of the diameter and the high flow, neither from the arterious side without sacrificing lobar branches.

## Case report

A 34-years-old Caucasian woman presented several episodes of paroxysmal supraventricular tachycardia (> 200 bpm) associated with dyspnoea, after the onset of post-pregnancy hypertension. She underwent Computed Tomography Angiography (CTA), spectral Doppler sonography and angiography which showed a renal arteriovenous fistula between the renal artery, at the hylum trifurcation point, and an extremely ectatic vein draining into the main renal vein of the right kidney, with two little areas of cortical infarction (25 ml total for a mean kidney volume around 900 ml) respectively on the anterior and inferior sides of the kidney.

**Fig. 1:**
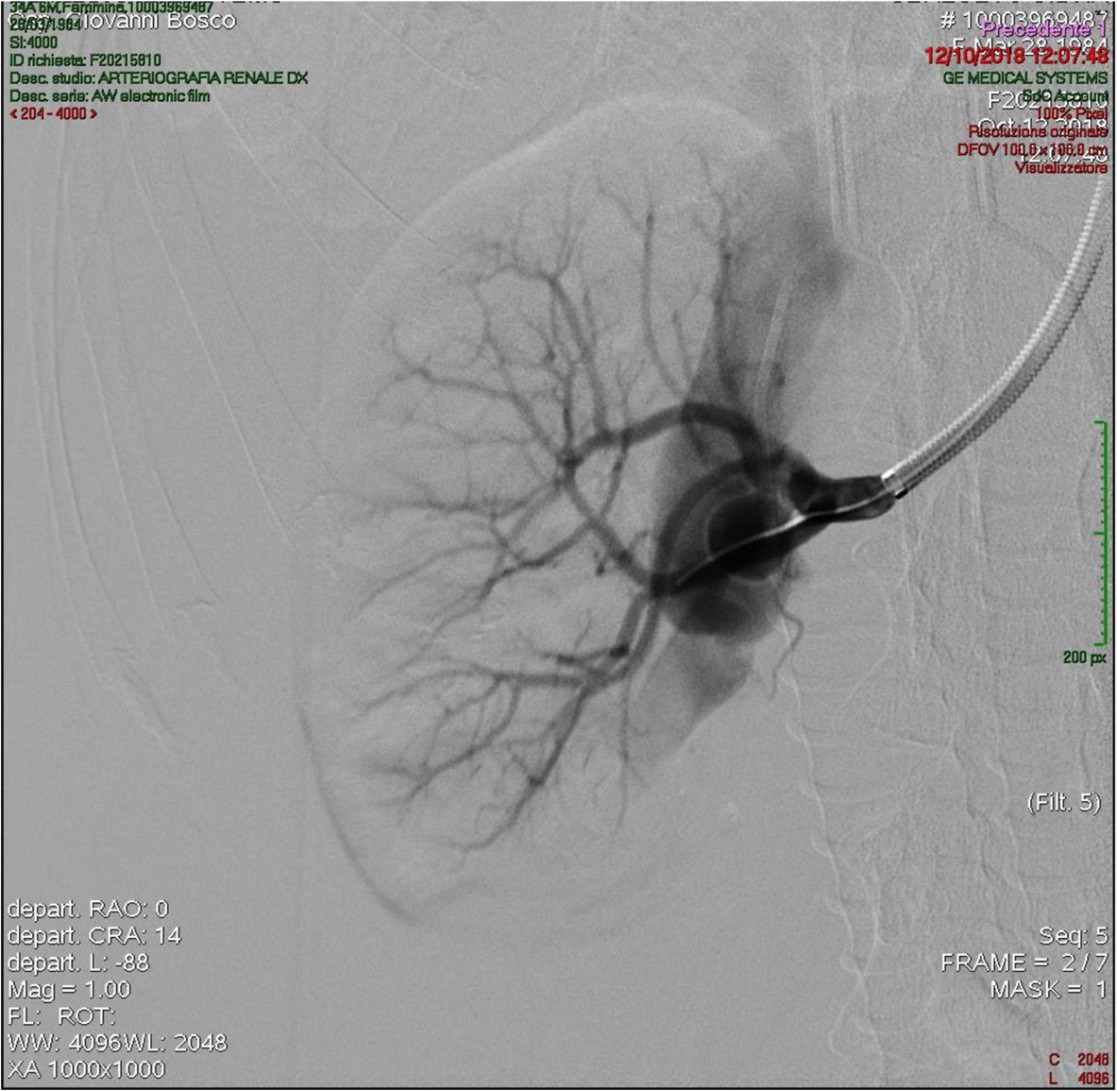
Angiography performed through a 7 F 70 cm long sheath showing the renal arteriovenous fistula between the renal artery, at the hylum trifurcation point, and an extremely ectasic vein draining into the main renal vein of the right kidney. The arteriovenous communication point was 3 mm wide

The flow of the shunt, measured with Doppler sonography, was more than 600 ml per minute. The arteriovenous communication point was 3 mm wide (Fig. 1).

The patient had no history of renal trauma or recent medical intervention with percutaneous instrumentation.

The renal function was normal (serum creatinine 0,8 mg/dl) with a slight hypokalemia (3,3 mEq/l) and coagulation parameters were within normal limits.

*We* chose as a device a 4 × 6 mm Amplatzer Vascular Plug II (AGA Medical Corporation).. We performed a simulation of the device placement using a plastic model.

The patient was informed about possible procedural complications, primarily the risk of massive acute right kidney ischemia due to device malpositioning and the consequent nephrectomy necessity, and she gave us consent.

The procedure was performed under general anesthesia. A short 4 F sheath was placed in the right femoral artery for blood pressure monitoring.

Selective right renal artery angiography was performed using a left brachial artery US-guided approach with a 7 F 70 cm long sheath (Flexor Check-Flo, Cook Medical), confirming the AVF beetween renal artery and vein at hylar site.

With a US-guided access in the right internal jugular vein, we reached the renal vein with a 6 F 45 cm long sheath (Flexor Check-Flo, Cook Medical).

From arterial side, a 260 cm long hydrophilic guidewire was passed through the site of communication between artery and vein, reaching inferior vena cava. That guidewire was caught with a 10 mm snare and brought outside the venous sheath.

**Fig. 2:**
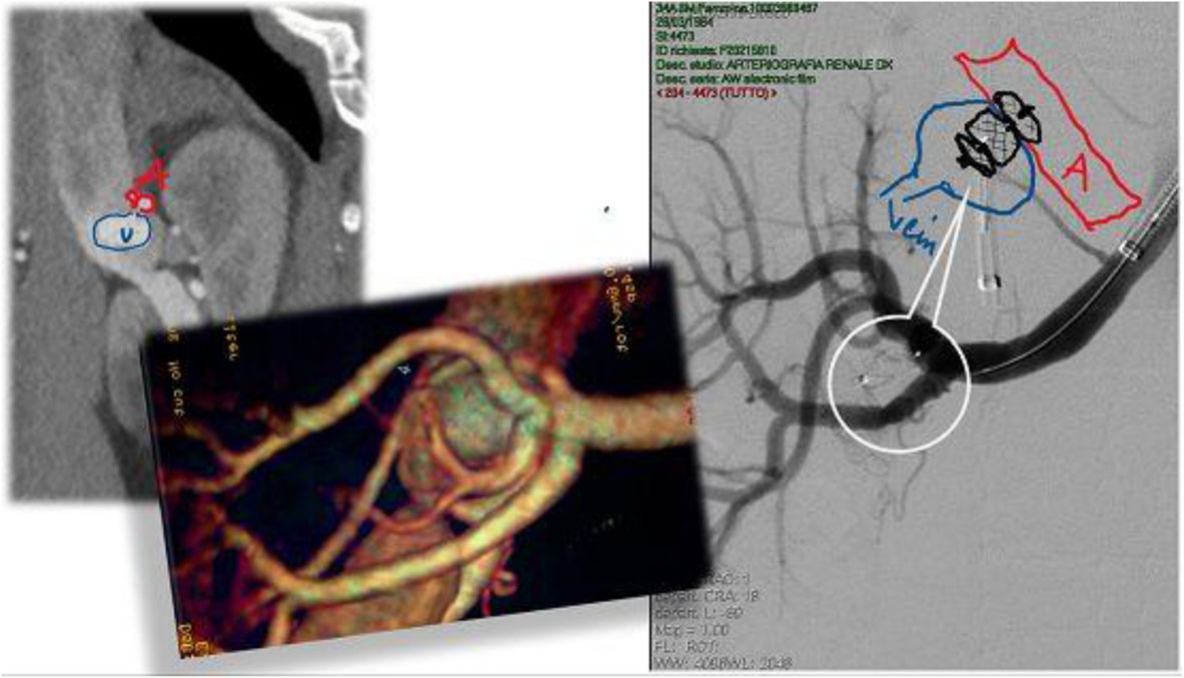
Left: CT and VR view of the communication point of the CRAVF. Right: Amplatzer Vascular Plug II released at extact level of the arteriovenous comunication

A 5 F JR4.0 cardiological guiding catheter (Launcher, Medtronic) was carried from the venous side on that guidewire, reaching the artery. After removing the wire, the Amplatzer Vascular Plug II was pushed through the guiding catheter into the artery side and the distal disc was opened in the artery lumen. Then the system (guiding catheter and vascular plug) was pulled-back at the exact level of the arteriovenous communication, anchoring only the distal disc against the arterial wall, inside arterial lumen, and releasing the other two proximal discs inside the venous ectasia (Fig. 2, right).

**Fig. 3:**
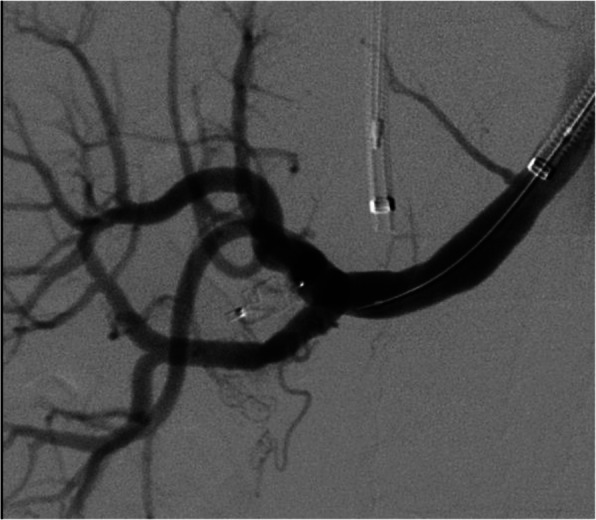
Control angiography showing the good procedural result

Immediate significant slowdown of the AVF was obtained, with a good renal vascularization, with contrast media filling the vein only in a late phase (Fig. 3).

As a precaution, a 6 × 40 mm balloon catheter (Armada, Abbott Vascular) was placed uninflated into the renal artery to be ready to manage any haemorragic complication.

**Fig. 4:**
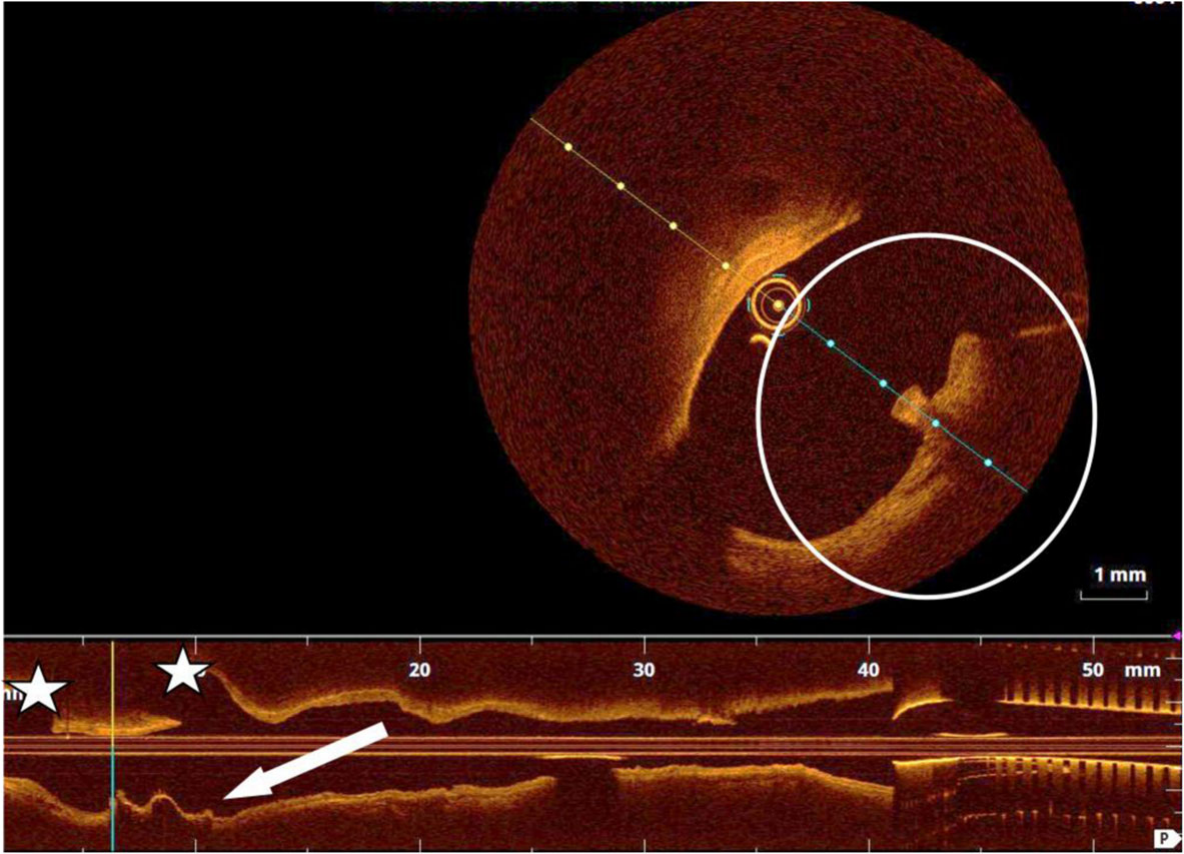
OCT multiplanar view of the arterial lumen showing the correct placement of the device (white circle and white arrow). The white stars identify the origin of two lobar branches

An optical coherence tomography (OCT) control was performed from the arterial side, demonstrating the good placement of the device (Fig. 4).

The patient underwent single antiplatelet therapy (acetylsalicylic acid 100 mg die) for 3 months.

The spectral Doppler sonography performed 3 days later confirmed the patency of right renal artery, showing a slight residual slow blood flow through the AVF.

The CTA performed two moths later demonstrated regular patency of the right renal artery and showed a slight residual blood flow through the AVF with a faint early enhancement of the renal vein, with no new ischaemic lesions.

The patient no longer suffered from tachicardia or dyspnoea and the renal function is normal (serum creatinine 0.76 mg/dl 1 year after the procedure).

The CTA control performed 1 year after the procedure showed a slight residual of the AVF, still asymptomatic, with a renal vein ectasia reduction.

## Conclusions

In this case, the placement of a covered stent in the arterial lumen was considered, but not feasible without sacrificing three arterial branches, due to their origin close to the arteriovenous communication point. For thisreason, it was necessary to close only the arteriovenous communication point to save all arterial supply. We also considered the option of placing spirals into the vein ectasia, but we excluded that in consideration of the high flow, with increased migration risk, and vein wall thinness, with possible rupture risk.

Other few cases presented the use of an Amplatzer Vascular plug for the embolization of kidney arteriovenous fistula (Campbell JE, Davis C, DeFade BP, Tierney JP, Stone PA., 2009; Perkov et al., 2013; Kayser & Schafer, 2013; Brountzos et al., 2009). In particular Perkov D et al. (Perkov et al., 2013) used a 12 mm Amplatzer Vascular Plug II released in the main artery feeding straight the arteriovenous fistula of the right kidney, while Kayser O et al. (Kayser & Schafer, 2013) deployed a 7 mm Amplatzer Vascular plug IV within the venous segment of the AVF and then they occluded the right renal artery with a 16 mm Amplatzer Vascular plug II.

In Perkov D case, the occluded artery feeded only the AVF, without parenchymal contributions. In Kayser O case, they performed a sort of endovascular nephrectomy.

Due to the anatomical arterial conformation, in our case it wasn’t possible to achieve arterial embolization without parenchymal damage. Considering the young age of the patient, we also aimed to avoid a proximal embolization of the main renal artery. So we released a 4 mm AVP II transversely to the artery, across the fistula point, with just one disk inside arterial lumen and the other two disks into the venous ectasia, like a patent foramen ovale (PFO) occluder.

Despite the fact that we didn’t achieve a complete technical success, we obtained good clinical success during 2 years follow up until now. In Literature is reported a 17% rate of “de novo” congestive heart failure in patients with hemodialysis AVF (Alkhouli et al., 2015). The mean flow in hemodialysis AVF is around 1.5 l/min. We lowered the patient’s shunt flow to less than 300 ml per minute, measured with Doppler sonography, lower than hemodialysis one. It looks unlikely that the patient would develop again a worsening in cardiac symptoms. Anyway our treatment didn’t exclude the possibility for further interventions, if needed.

## Data Availability

Not applicable.

## Abbreviations

RAVF: Renal arteriovenous fistula
CTA: Computed Tomography Angiography
HOS: High output cardiac state
OCT: Optical coherence tomography
PFO: Patent foramen ovale
TAVI: Transcatheter aortic valve implantation
